# Cadmium Exposure Disrupts Uterine Energy Metabolism and Coagulation Homeostasis During Labor in Institute of Cancer Research Mice: Insights from Transcriptomic Analysis

**DOI:** 10.3390/metabo15050339

**Published:** 2025-05-20

**Authors:** Yueyang Wang, Yichen Bai, Yi Wang, Yan Cai

**Affiliations:** 1 Department of Obstetrics, The Fourth Affiliated Hospital of Harbin Medical University, Harbin 150001, China; yywang@hrbmu.edu.cn; 2 College of Animal Science and Technology, Northeast Agricultural University, Harbin 150030, China; baiyichen1621@163.com (Y.B.); s230502054@neau.edu.cn (Y.W.)

**Keywords:** cadmium, uterus, gene, transcriptomic analysis

## Abstract

**Background:** Cadmium (Cd) is a highly toxic heavy metal. There are very few studies about the effects of Cd on reproductive health and metabolism, and even fewer on metabolic disorders in the uterus of mice in labor. This study is the first to establish a model of Cd exposure in the uterus of laboring mice and investigate the underlying metabolic mechanisms through transcriptomic analysis. **Methods:** Pregnant mice received intraperitoneal injections of CdCl_2_ (1.5 mg/kg) on gestational days 12.5, 14.5, and 16.5 were set up as the experimental group (Cd group), and pregnant mice injected with saline were set up as the control group (CT group). A total of 738 differentially expressed genes (DEGs) were screened using DESeq2 software, including 326 upregulated genes and 412 downregulated genes. **Results:** Through enrichment databases including the KEGG, GO, Reactome, and PANTHER, we identified 76 metabolism-related DEGs and performed protein–protein interaction (PPI) network analysis. The PPI results were visualized using Cytoscape software and further analyzed, with 18 hub genes (maximum clique centrality score > 10) identified through the MCC algorithm of the Cytohubba plugin. The results showed that the highest-scoring hub genes included *mt-Co2*, *mt-Co3*, *mt-Atp6*, *mt-Atp8*, *mt-Nd3*, and *mt-Nd4l*, which are involved in mitochondrial energy metabolism. The remaining lower-scoring hub genes were primarily associated with coagulation processes. Pathway analysis revealed hub genes predominantly involved in oxidative phosphorylation, complement and coagulation cascades, the cGMP-PKG signaling pathway, and thermogenesis. **Conclusion:** This study successfully established a Cd exposure-induced uterine injury model, providing valuable references for human reproductive health research.

## 1. Introduction

Cadmium (Cd) is widespread in the environment as a heavy metal contaminant [1,2,3] and has a half-life of 10–30 years in the human body after entry through the food chain, with significant bioaccumulation [4]. Chronic exposure to Cd has been associated with various adverse health outcomes, including cardiovascular diseases [5], as well as liver, kidney, and bone damage [6]. In severe cases, Cd exposure has been implicated in carcinogenesis [7]. The reproductive toxicity of Cd has received much attention in recent years, with studies finding that low doses of Cd are sufficient to adversely affect the reproductive health of human males and females [8]. It has been reported that the testis is highly sensitive to Cd toxicity, and Cd causes severe structural disruption to supporting cells (SCs), seminiferous tubules, and the blood–testis barrier, leading to sperm loss [9]. Similarly, Cd exposure also poses a risk to female reproductive health [8,10]. In 1999, Toyama Medical University Hospital in Japan found that Cd exposure during women’s pregnancy not only increased preterm birth rates but also significantly reduced the weight and height of infants [11]. In a recent study, Zhang et al. established a uterus-specific Cd accumulation mouse model and verified that Cd-accumulating mice had increased numbers of absorbed fetuses and defective decidualization [12]. Cd-induced uterine damage can affect embryonic development and the labor process [13,14]. However, research on the effect of Cd toxicity on the laboring uterus remains limited, and the relative metabolic mechanisms are unknown. So, we hypothesize that Cd exposure may have a negative impact on the laboring uterus; in addition, we know that ICR mice are model animals for studies on human reproductive problems [15,16]. Thus, in the present study, ICR mice were used as test animals to explore the Cd poisoning effect on the laboring uterus.

Successful labor depends on rhythmic contractions of the myometrium [17] and rapid hemostasis after placental detachment [18], both of which are intricately regulated by energy metabolism and the coagulation–fibrinolytic system [19]. However, disturbances in energy metabolism can lead to abnormal contractions of the uterine myometrium, which in turn can trigger obstructed labor [20]. Oxidative phosphorylation serves as a central pathway of energy metabolism, responsible for the transfer of electrons in the respiratory chain as well as ATP synthesis [21]. During labor, oxidative phosphorylation plays a crucial role in supplying ATP to the myometrium, thereby ensuring the maintenance of rhythmic contractions and adequate contraction intensity [22]. In addition, the oxidative phosphorylation process is dependent on respiratory chain complexes for providing proton motive force, and *mt-Nd3* and *mt-Nd4l* are responsible for encoding respiratory chain complex I [23,24], which catalyzes the dehydrogenation of NADH and establishes the proton gradient, thereby offering an energetic basis for ATP synthesis [25]. In their study of mouse germinal vesicle (GV) stage oocytes, Zhang et al. discovered that expression levels of *mt-Nd3* and *mt-Nd4l* were decreased, and this decrease was associated with the disruption of oxidative phosphorylation and mitochondrial respiratory chain function [26]. Furthermore, *mt-Co2* and *mt-Co3* are responsible for encoding core subunits of respiratory chain complex IV, which is essential for both ATP synthesis-coupled electron transport and mitochondrial electron transport [27,28]. In primary human hepatocytes, it was found that valproic acid (VPA) decreased the expression levels of *mt-Co2* and *mt-Co3*, and in turn, a sustained decrease in ATP production was observed [29]. During the ATP synthesis process, *mt-Atp6* and *mt-Atp8* play a crucial role in the synthesis and assembly of mitochondrial ATP synthase (respiratory chain complex V) [30,31]. According to the study by Xiang et al., the downregulation of *MT-ATP6* in human chondrocytes leads to the inhibition of oxidative phosphorylation and affects ATP production [32]. In addition, Yan et al. found that a low-protein diet leads to the downregulation of MT-ATP8 in the longissimus dorsi muscle of weaned piglets, thereby resulting in the inhibition of the oxidative phosphorylation pathway and affecting growth performance [33]. However, the effects of heavy metal poisoning on the above mitochondrial energy metabolism-related genes have not been reported; therefore, we wanted to investigate whether Cd poisoning causes disorders of mitochondrial energy metabolism in the laboring uterus.

Coagulation plays an indispensable role in the process of labor. At the end of labor, the uterus requires prompt hemostasis to prevent infection and inflammation due to placental detachment [34]. The vascular endothelium is the primary target of Cd toxicity [35]; Cd uptake induces endothelial damage, promotes platelet activation, increases fibrin thickness, and triggers pro-thrombotic mechanisms in human endothelial cells, ultimately leading to coagulation disorders [36]. Therefore, Cd exposure may significantly disrupt both uterine energy metabolism and coagulation processes during labor, potentially increasing the risk of labor complications. Worldwide, coagulation disorders during labor contribute to 25% of the annual maternal hemorrhage-related deaths [37]. *Serpind1* is responsible for encoding Heparin cofactor II (HCII), a powerful thrombin inhibitor [38]. It has been reported that, compared with wild-type mice, HCII-deficient mice develop carotid thrombosis more rapidly following endothelial oxidative damage [39]. Another study showed that silencing HCII and targeting it to the liver significantly improved the coagulation capacity of hemophilia mice [40]. *Fgg* is responsible for encoding plasma fibrinogen, which forms a stable fibrin mesh structure via thrombin during coagulation and participates in hemostasis and wound repair [41]. A cross-sectional study conducted in Copenhagen, Denmark, revealed that anabolic–androgenic steroid (AAS) users exhibit significantly elevated fibrinogen levels and reduced fibrin clot solubility. Prolonged AAS use induces sustained hypercoagulability, thereby elevating thrombotic risk [42]. *Kng1* encodes high-molecular-weight kininogen (HK), a major player in the endogenous coagulation pathway, and is directly involved in platelet aggregation and thrombosis under the action of thrombin [43]. It has been reported that the deletion of the mouse kininogen gene (*Kng1*) causes the loss of plasma kininogen and leads to delayed thrombosis. Additionally, the knockout of the *Kng1* gene results in a prolonged arterial clotting time in mice [44]. Another study found that KNG deficiency alleviated cerebral artery occlusion and reduced thrombosis in ischemic vessels in mice [45]. It is noteworthy that the effects of heavy metal poisoning on the *Serpind1* and *Kng1* genes have not been reported, so we wanted to investigate whether Cd poisoning causes uterus coagulation disorders during labor.

Transcriptomics analysis has many advantages in revealing molecular mechanisms, and the main transcriptome sequencing technologies currently available include microarray and second-generation sequencing [46]. Among these, RNA sequencing (RNA-Seq), a transcriptomics approach based on next-generation sequencing technology, is widely utilized for gene expression analysis and the discovery of novel RNA species [47]. Transcriptomic analysis techniques can also be used to help discover metabolic mechanisms [48]; recently, some researchers used transcriptomic techniques to find that gallate (EGCG) can alleviate amino acid metabolic disorders caused by Mn through the miR-9-5p/got1 axis [49]. Similarly, Mao et al. found that dysregulated metabolism and metabolic disorders were associated with a high-salt diet and that the high-salt diet inhibited hepatic lipogenesis through transcriptomic analysis [50]. In addition, protein–protein interaction networks (PPIs) can be constructed using differentially expressed genes (DEGs) obtained from transcriptome sequencing [19], and Cytoscape software can visualize PPIs and can be used to help identify important nodes in PPIs using the maximal clique centrality (MCC) algorithm via the Cytohubba plug-in [51,52]. The MCC algorithm evaluates the importance of a node in the network by counting the size and number of the largest groups in which the node participates, and a node with a higher score is in a more pivotal position in the whole network, which can help researchers to narrow down the scope of the study and provide a direction for research [53,54]. Therefore, we wanted to investigate whether Cd contributes to energy metabolism disorders and coagulation disorders during labor in ICR mice from a big data perspective.

Despite these advances, studies on the effects of Cd toxicity on metabolic mechanisms in the laboring uterus are still lacking. To further reveal the mechanism of Cd toxicity, we established a model of Cd exposure in the uteri of mice in labor for the first time and preliminarily identified the metabolic mechanisms and hub genes mainly affected by Cd through protein–protein interaction network analysis of DEGs in the transcriptome and their visualization. The present study aimed to further assess the toxic effects of Cd exposure on the uteri of mice in labor and provide novel information on the mechanisms of reproductive toxicity caused by Cd.

## 2. Materials and Methods

### 2.1. Chemicals

Cadmium chloride (CdCl_2_, purity ≥ 99%) was purchased from Sigma Chemical Co. (St. Louis, MO, USA) and dissolved in distilled water. We used 1.25% Tribromoethanol for ready use (TBE, MA0478-2, 30 μL/g) that was provided by Meilun Biotechnology Co., Ltd. (Dalian, China) for the anesthesia of mice during labor. Phosphate-buffered saline (PBS) was bought from Beyotime Biotech Inc. (Shanghai, China). Total RNA was extracted with TRIzol Reagent from Takara Bio Inc. (Kusatsu, Japan).

### 2.2. Animals

SPF-grade, healthy female and male ICR mice of reproductive age (6 weeks old, 25–28 g) were purchased from Liaoning Changsheng Biotechnology Co., Ltd. (Changchun, China). The mice were housed under a 12/12 h light–dark cycle, with free access to food and water, at a controlled temperature of 23–26 °C and humidity of 50–60%. All purchased mice underwent a one-week acclimatization period prior to the experiments being conducted. The animal experiments were conducted in compliance with the ethical guidelines set by the Institutional Animal Ethics Committee of the Fourth Clinical Hospital of Harbin Medical University (Approval No.: 2022-DWSYLLCZ-58).

### 2.3. Animal Treatment

Mating was conducted overnight at a female-to-male ratio of 2:1. The day the copulation plug was detected was marked as gestation day (GD) 0.5. The pregnant mice were then randomly assigned to two groups (*n* = 8 per group) as follows: (1) control group (CT); (2) Cd group (Cd) (CdCl_2_, 1.5 mg/kg body weight [bw]). The oral LD_50_ value of CdCl_2_ for mice is 109 mg/kg bw [55]. In this study, a test dose of 1.5 mg/kg bw CdCl_2_ was used, which is less than 1/50 of the LD_50_ value indicated for mice and was selected based on prior knowledge [56,57,58,59]. CdCl_2_ was dissolved in PBS at a concentration of 0.3 mg/mL and administered via intraperitoneal (i.p.) injection on GD12.5, GD14.5, and GD16.5. Mice in the CT group received an equivalent volume of PBS based on body weight (5 mL/kg bw) at the corresponding time points.

The physiological state of pregnant mice was closely monitored starting from gestational day 19 (GD19), with observations conducted hourly to check for reduced activity and the onset of uterine contractions. After the first pup was delivered, the pregnant mice were injected with an anesthetic, 1.25% Tribromoethanol (30 µL/g), to facilitate tissue collection.

### 2.4. Transcriptomics Analysis

Total RNA was extracted from the uteri of mice giving birth using the TRIzol method, and high-quality RNA (RIN/RQS > 7, OD260/280: 1.8–2.2, OD260/230 2.0–2.2) was selected for the creation of cDNA libraries using Agilent2100/Labchip, agarose gel electrophoresis, and Nanodrop. Raw reads were quality-filtered using Fastp V0.20. Clean data were compared to the reference genome using Hisat2 software, selecting the reference genome and annotation files of mice on NCBI https://www.ncbi.nlm.nih.gov/datasets/genome/GCF_000001635.27/ (accessed on 14 March 2024), using default parameters for comparison, and then evaluating the comparison of reads (read segments) obtained from sequencing. Using featureCounts v1.5.0 software, the number of reads for each gene was calculated from the SAM comparison file generated by Hisat comparison and the GTF annotation file of the genome, and then, the FPKM value of the gene was calculated according to the length of the exon using the above formula to indicate gene expression. Differences were screened using DESeq2 software(R package version 1.10.1). The screening criteria were *p*-value < 0.05 and|log2FC| ≧ 1.

### 2.5. Gene Ontology and Pathway Enrichment

Gene ontology (GO) enrichment analysis was performed for differentially expressed genes using the clusterProfiler package in R (version 1.10.1). The GO term screening condition for significant enrichment was a *p*-value of less than 0.05 for the hypergeometric distribution test. Kyoto encyclopedia of genes and genomes (KEGG) enrichment analysis was also performed using the clusterProfiler package. The KEGG pathway screening condition for significant enrichment was a *p*-value of less than 0.05 for the hypergeometric distribution test. In addition, to further illustrate the signaling pathways associated with DEGs, Reactome and PANTHER pathway enrichment analyses were performed using KOBAS software (version 3.0), http://bioinfo.org/kobas (accessed on 2 March 2025) [60]. Bioinformatic analysis was performed using the OmicStudio tools at https://www.omicstudio.cn/tool (accessed on 13 March 2025).

### 2.6. Protein–Protein Interaction Network and Identification of Hub Genes

To further understand the biological processes and molecular mechanisms of DEGs, the protein–protein interaction networks (PPIs) of DEGs were constructed using the STRING database https://cn.string-db.org/ (accessed on 3 March 2025), and the PPI results were visualized using Cytoscape software (version 3.10.3). To identify the hub genes involved in the mechanism of Cd-induced toxicity in the uteri of mice, the maximum group centrality score of the top 20 metabolically related DEGs was calculated using the MCC algorithm of the CytoHubba plugin, and the genes with scores greater than 10 were selected through the results [61].

## 3. Results

### 3.1. RNA-Seq and Differentially Expressed Genes

We constructed a total of six cDNA libraries with reads ranging from 60,731,292 to 89,369,498 for each transcriptome library, and a total of 463,330,824 pure reads were obtained through screening, of which 211,646,210 were for the CT group and 251,684,614 for the Cd group (Table 1).

**Figure 1:**
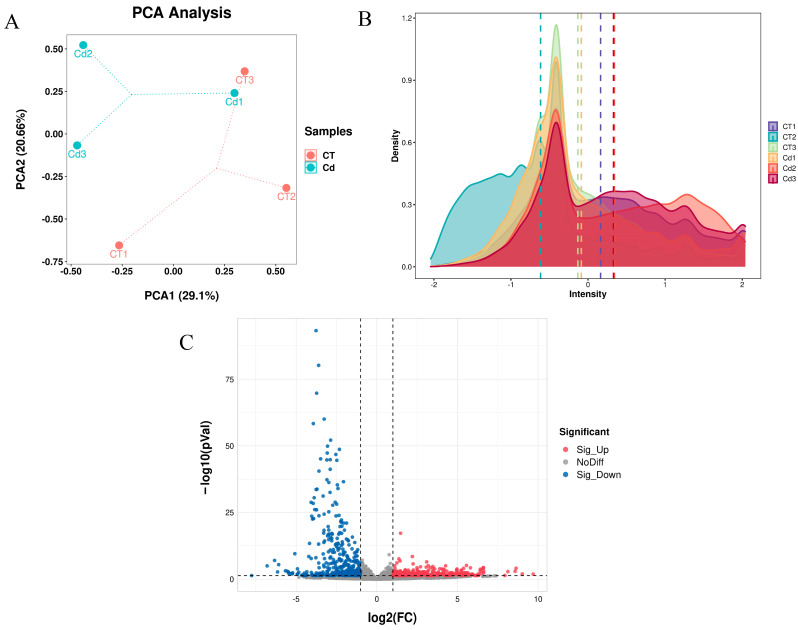
The gene landscape in the uteri of mice in labor under Cd exposure. (**A**) Principal component analysis (PCA) of the samples from the Cd and CT groups, with the samples showing variability in distance in space. (**B**) The density graph demonstrates the distribution of gene expression intensity in different sample groups. (**C**) Volcano plot for screening differentially expressed genes (DEGs) between groups.

A total of 27,688 genes were identified in the Cd group versus the CT group (Supplementary File S1), with PCA and expression density shown in Figure 1A,B, respectively; the volcano plot in Figure 1C shows the differentially expressed genes (DEGs) in the uteri of the Cd group compared to the CT group, with a total of 738 DEGs identified, of which 326 genes were upregulated and 412 genes were downregulated (Supplementary File S2).

### 3.2. Kyoto Encyclopedia of Genes and Genomes Enrichment

**Figure 2:**
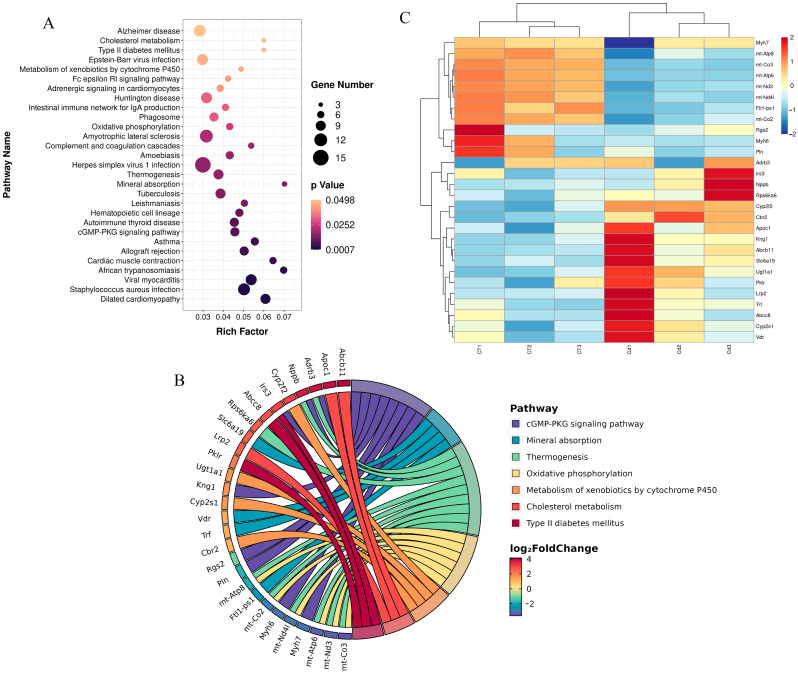
(**A**) Kyoto encyclopedia of genes and genomes (KEGG) enrichment for the DEGs in the uteri of mice in labor under Cd exposure. (**B**) Metabolism-related pathways in KEGG enrichment and DEGs included in the pathways. (**C**) Heatmap of the expression of DEGs associated with metabolism.

KEGG enrichment analysis was performed using the cluster profiler package of R. A total of 29 pathways were significantly enriched (Figure 2A) (*p* < 0.05), of which a total of 7 pathways were related to metabolic pathways (Figure 2B), accounting for 24.14% of all pathways. These include oxidative phosphorylation, the cGMP-PKG signaling pathway, cholesterol metabolism, mineral absorption, thermogenesis, the metabolism of xenobiotics by cytochrome P450, and type II diabetes mellitus.

A total of 28 DEGs were enriched in these seven pathways (Figure 2C), including upregulated ones—such as *Cyp2f2*, *Cbr2*, *Apoc1*, *Abcb11*, *Slc6a19*, *Kng1*, *Nppb*, *Cyp2s1*, *Ugt1a1*, *Irs3*, *Pklr*, *Rps6ka6*, *Vdr*, *Lrp2*, *Adrb3*, *Trf*, and *Abcc8*—and downregulated ones—such as *mt-Co3*, *mt-Atp6*, *Ftl1-ps1*, *mt-Nd3*, *mt-Nd4l*, *mt-Co2*, *mt-Atp8*, *Myh6*, *Pln*, *Myh7*, and *Rgs2*.

### 3.3. Gene Ontology Enrichment

**Figure 3:**
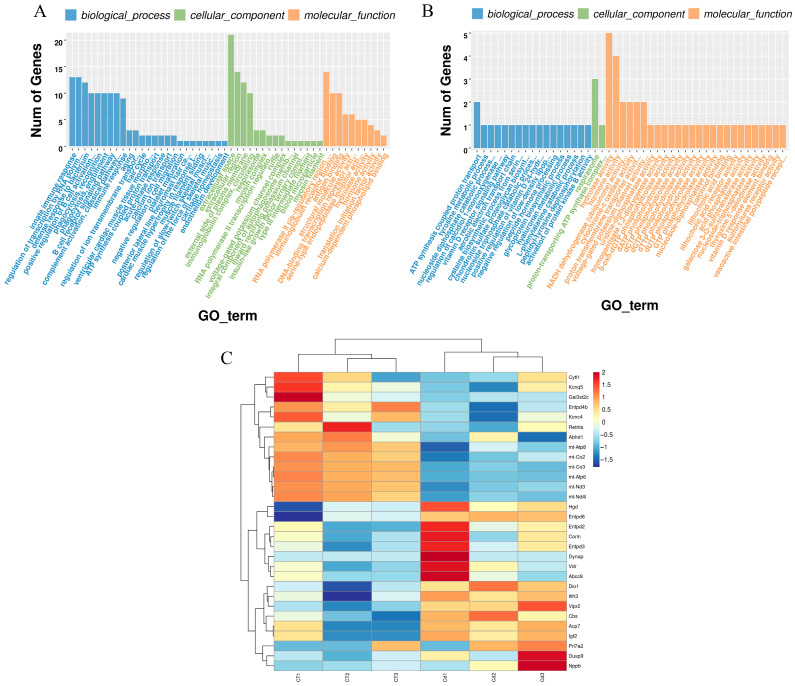
(**A**) Gene ontology (GO) enrichment for DEGs in the uteri of mice in labor under Cd exposure. (**B**) Metabolism-related pathways in GO enrichment and DEGs included in the pathways. (**C**) Heatmap of the expression of DEGs associated with metabolism.

Gene ontology (GO) enrichment analyses of differentially expressed genes were performed using the clusterProfiler in R, and a total of 178 terms were significantly enriched (*p* < 0.05) (Figure 3A and Supplementary File S3).

Forty-five of the GO terms were related to metabolic pathways (Figure 3B), accounting for 25.28% of all entries, including 8-oxo-dGTP, dATP, dCTP, dGTP, dTTP, dUTP and GTP phosphohydrolase activity, acid phosphatase activity, activation of protein kinase B activity, aerobic electron transport chain, ATP synthesis-coupled proton transport, calcitriol binding, the chondroitin sulfate proteoglycan biosynthetic process, the cysteine biosynthetic process from serine, cytochrome-c oxidase activity, galactose 3-O-sulfotransferase activity, the glycoprotein biosynthetic process, homogentisate 1,2-dioxygenase activity, hormone activity, the hyaluronan metabolic process, hydrolase activity, lithocholic acid binding and receptor activity, the L-phenylalanine catabolic process, NADH dehydrogenase (ubiquinone) activity, the negative regulation of low-density lipoprotein particle clearance, the nucleoside diphosphate catabolic process, the nucleoside triphosphate catabolic process, nucleoside-diphosphatase activity, nucleoside-triphosphatase activity, peptide hormone processing, peptidyl-tyrosine dephosphorylation, the positive regulation of vitamin D 24-hydroxylase activity, proton transmembrane transporter activity, the proton-transporting ATP synthase complex, coupling factor F(o), the regulation of calcidiol 1-monooxygenase activity, respirasome, sulfonylurea receptor activity, thyroxine 5’-deiodinase activity, the tyrosine metabolic process, uridine-diphosphatase activity, vasoactive intestinal polypeptide receptor activity, the vitamin D receptor signaling pathway, vitamin D response element binding, and voltage-gated potassium channel activity.

A total of 30 DEGs were enriched in these 45 terms (Figure 3C), including upregulated ones—such as *Acp7*, *Cbs*, *Corin*, *Dio1*, *Itih3*, *Dusp9*, *Igf2*, *Vipr2*, *Nppb*, *Hgd*, *Entpd3*, *Entpd2*, *Entpd8*, *Vdr*, *Prl7a2*, *Dynap*, and *Abcc8*—and downregulated ones—such as *mt-Co3*, *mt-Atp6*, *mt-Nd3*, *mt-Nd4l*, *mt-Co2*, *Entpd4b*, *mt-Atp8*, *Abhd1*, *Retnla*, *Kcnq5*, *Gal3st2c*, *Kcnc4*, and *Cytl1*.

### 3.4. Reactome Enrichment

**Figure 4:**
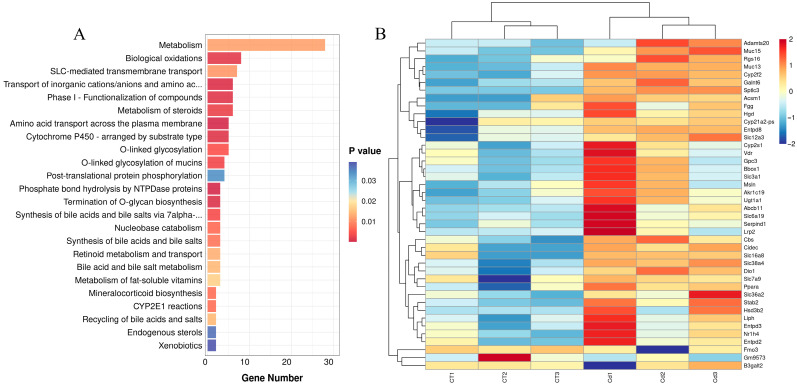
(**A**) Reactome enrichment for the DEGs in the uteri of mice in labor under Cd exposure. (**B**) Heatmap of the expression of DEGs associated with metabolism.

A total of 43 pathways were significantly enriched (*p* < 0.05) in the Reactome enrichment analysis (Supplementary File S4), of which 24 pathways were metabolism-related (Figure 4A), accounting for 55.81% of all pathways, which shows that the metabolism pathway was the most enriched. Among the pathways related to metabolism were amino acid transport across the plasma membrane, bile acid and bile salt metabolism, biological oxidations, CYP2E1 reactions, cytochrome P450—arranged by substrate type, endogenous sterols, metabolism, the metabolism of fat-soluble vitamins, the metabolism of steroids, mineralocorticoid biosynthesis, nucleobase catabolism, O-linked glycosylation, the O-linked glycosylation of mucins, phase I—the functionalization of compounds, phosphate bond hydrolysis by NTPDase proteins, post-translational protein phosphorylation, the recycling of bile acids and salts, retinoid metabolism and transport, SLC-mediated transmembrane transport, the synthesis of bile acids and bile salts, the synthesis of bile acids and bile salts via 7alpha-hydroxycholesterol, the termination of O-glycan biosynthesis, the transport of inorganic cations/anions, and amino acids/oligopeptides, and xenobiotics.

A total of 42 DEGs were enriched in these 24 pathways (Figure 4B), the upregulated of which included *Galnt6*, *Slc38a4*, *Muc13*, *Cyp2f2*, *Cidec*, *Abcb11*, *Rgs16*, *Liph*, *Nr1h4*, *Cbs*, *Slc6a19*, *Dio1*, *Stab2*, *Gpc3*, *Akr1c19*, *Hgd*, *Cyp2s1*, *Cyp21a2-ps*, *Ugt1a1*, *Entpd3*, *Slc7a9*, *Entpd2*, *Bbox1*, *Hsd3b2*, *Adamts20*, *Entpd8*, *Acsm1*, *Serpind1*, *Vdr*, *Muc15*, *B3galt2*, *Sptlc3*, *Lrp2*, *Ppara*, *Slc3a1*, *Slc36a2*, *Msln*, *Fgg*, *Slc16a8*, and *Slc12a3*, and the downregulated of which included *Fmo3* and *Gm9573*.

### 3.5. PANTHER Enrichmet

PANTHER enrichment analysis highlighted that Cd-induced DEGs may be involved in cytoskeletal regulation by Rho GTPase, insulin/IGF pathway–mitogen-activated protein kinase kinase/MAP kinase cascade, and the nicotinic acetylcholine receptor signaling pathway. Insulin/IGF pathway–mitogen-activated protein kinase kinase/MAP kinase cascade is most relevant to the metabolic process and that *Igf2* and *Irs3* genes in DEGs were enriched in this pathway and both were upregulated (Table 2).

### 3.6. Integration of the Protein–Protein Interaction Network

After the above pathway enrichment and database annotation, it was found that the metabolism-related pathways and terms accounted for 24.14%, 25.28%, 55.81%, and 33.3% of KEGG, GO, Reactome, and PANTHER enrichment, respectively, which indicated that Cd induction mainly affected the metabolic mechanism.

**Figure 5:**
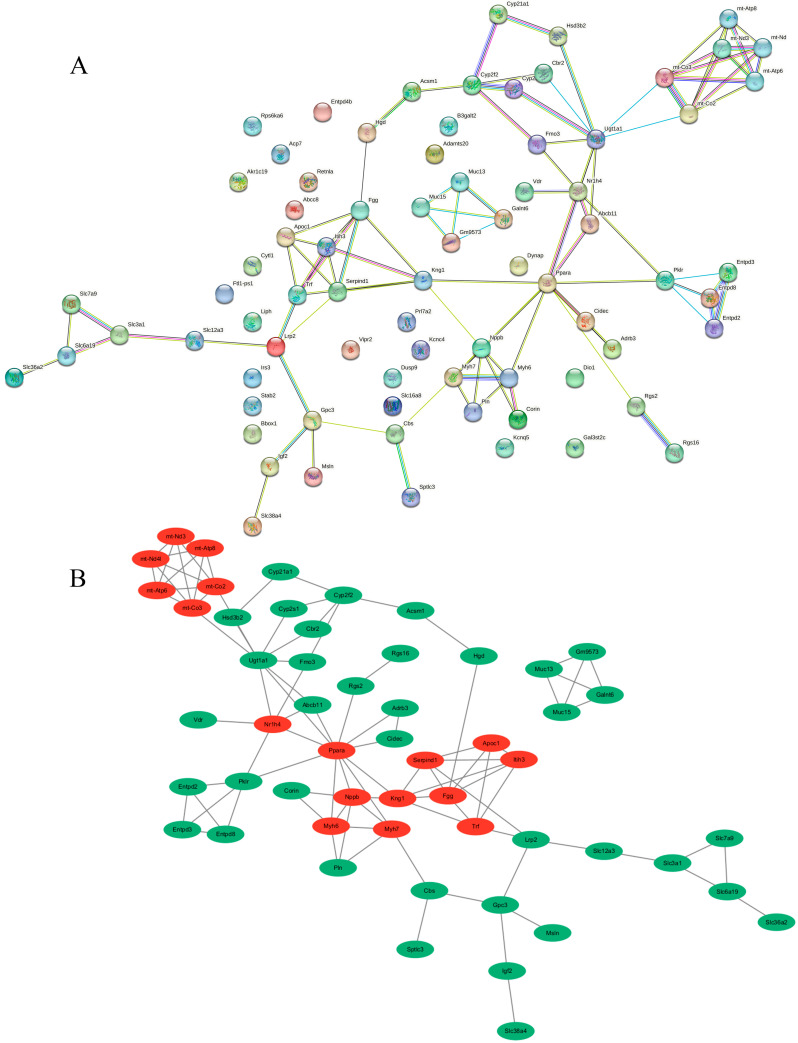
(**A**) Protein–protein interaction (PPI) network analysis for DEGs related to metabolism (The color of nodes represents the type of protein, and the color of connections represents the interaction relationship between proteins). (**B**) Visualization of PPIs by Cytoscape software and labeling of hub genes in topological networks (The green ellipses represent genes in the protein-protein interaction network, and the red ellipses denote hub genes).

After counting, we found a total of 76 DEGs related to metabolic pathways, including *Abcb11*, *Abcc8*, *Acp7*, *Adrb3*, *Adrb3*, *Akr1c19*, *Apoc1*, *B3galt2*, *Bbox1*, *Cbr2*, *Cbs*, *Cidec*, *Corin*, *Cyp2f2*, *Cyp2s1*, *Cyp21a2-ps*, *Dio1*, *Dusp9*, *Entpd2*, *Entpd3*, *Entpd8*, *Fgg*, *Galnt6*, *Gpc3*, *Hgd*, *Hsd3b2*, *Irs3*, *Itih3*, *Kng1*, *Liph*, *Lrp2*, *Msln*, *Muc13*, *Muc15*, *Nr1h4*, *Nppb*, *Pklr*, *Ppara*, *Prl7a2*, *Retnla*, *Rgs16*, *Rps6ka6*, *Serpind1*, *Slc12a3*, *Slc16a8*, *Slc3a1*, *Slc36a2*, *Slc38a4*, *Slc6a19*, *Slc7a9*, *Stab2*, *Trf*, *Ugt1a1*, *Vdr*, *Vipr2*, *Abhd1*, *Adamts20*, *Cytl1*, *Entpd4b*, *Fmo3*, *Ftl1-ps1*, *Gal3st2c*, *Gm9573*, *Igf2*, *Kcnc4*, *Kcnq5*, *mt-Atp6*, *mt-Atp8*, *mt-Co2*, *mt-Co3*, *mt-Nd3*, *mt-Nd4l*, *Myh6*, *Myh7*, *Pln*, *Rgs2,* and *Sptlc3.* To delineate the functional interplay of metabolism-associated genes, we constructed a protein interaction network using the STRING database (Figure 5A).

The resultant PPI network was visualized via Cytoscape (v3.10.3), followed by topological analysis employing the maximal clique centrality (MCC) algorithm in CytoHubba. The MCC algorithm identified a total of 18 hub genes with scores greater than 10 (Supplementary File S5), including *Fgg*, *Itih3*, *Ppara*, *Nppb*, *Kng1*, *Serpind1*, *Trf*, *Ugt1a1*, *Apoc1*, *Nr1h4*, *mt-Co2*, *mt-Co3*, *mt-Atp6*, *mt-Atp8*, *mt-Nd3*, *mt-Nd4l*, *Myh6,* and *Myh7.* PPI visualization showed two distinct topological clusters, a bimodal structure that may suggest a dual pathogenic mechanism (Figure 5B).

### 3.7. Hub Genes and Their Functions

**Figure 6:**
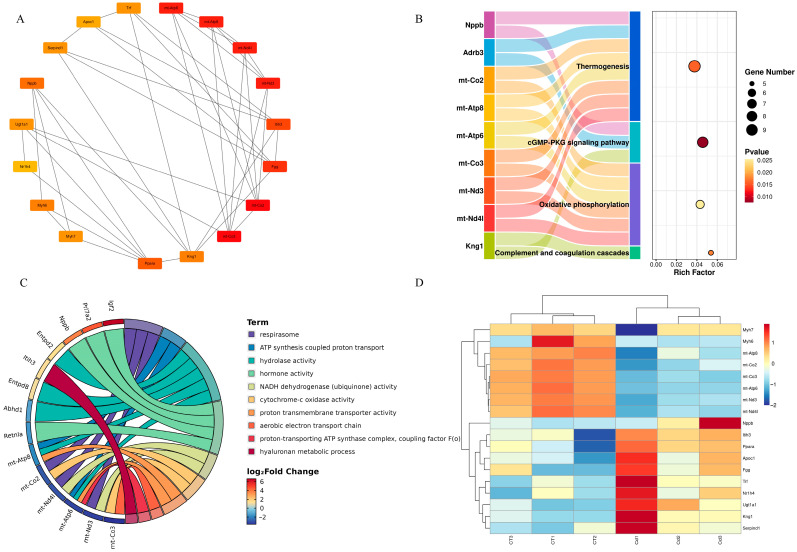
(**A**) Hub genes related to metabolism in the uteri of mice in labor under Cd exposure. (**B**) Hub gene enrichment in the KEGG pathway. (**C**) Annotation of hub genes in GO enrichment. (**D**) Heatmap of the expression of hub genes associated with metabolism.

The hub genes identified by the MCC method are shown in Figure 6A. In addition, pathway analysis revealed that of the 18 hub genes derived from the MCC algorithm, the main KEGG pathways involved were oxidative phosphorylation, complement and coagulation cascades, the cGMP-PKG signaling pathway, and thermogenesis (Figure 6B).

The main annotated GO terms of hub genes are respirasome, ATP synthesis-coupled proton transport, hydrolase activity, hormone activity, NADH dehydrogenase (ubiquinone) activity, cytochrome-c oxidase activity, proton transmembrane transporter activity, aerobic electron transport chain, the proton-transporting ATP synthase complex, coupling factor F(o), and the hyaluronan metabolic process (Figure 6C). In addition, upregulated hub genes included *Fgg*, *Itih3*, *Ppara*, *Nppb*, *Kng1*, *Serpind1*, *Trf*, *Ugt1a1*, *Apoc1,* and *Nr1h4*, while downregulated ones included *mt-Co2*, *mt-Co3*, *mt-Atp6*, *mt-Atp8*, *mt-Nd3*, *mt-Nd4l*, *Myh6*, and *Myh7* (Figure 6D).

## 4. Discussion

### 4.1. Metabolism Was the Primary Mechanism of Cd Poisoning in the Uteri of ICR Mice in Labor

The uterus, as the central organ of the female reproductive system, plays a key role in the physiological process of reproduction [62]. The uterus maintains reproductive endocrine balance through dynamic regulation of the menstrual cycle [63] and provides a suitable microenvironment for embryo implantation and development [64]. The realization of the above physiological functions is dependent on the careful regulation of metabolic homeostasis in the organism [65,66,67]. Previous studies have reported that heavy metal poisoning causes metabolic disorders [49,68,69]; however, the effects of cadmium (Cd) poisoning on the laboring uterus have not been reported yet, particularly from the perspective of metabolism-related research. Therefore, we established a model of Cd exposure in the uteri of mice in labor for the first time. In this study, a total of 738 differentially expressed genes were identified by transcriptomic analysis techniques. Enrichment analysis by KEGG, GO, Reactome, and PANTHER revealed that metabolism-related pathways or terms accounted for 24.14%, 25.28%, 55.81%, and 33.3% of the total number, respectively, suggesting that metabolic disorders are the main mechanism of Cd poisoning, so we would like to further investigate the mechanism of Cd poisoning in the uteri of laboring mice from a metabolic perspective.

### 4.2. KEGG and GO Enrichment Analysis Further Confirmed That Mitochondrial Energy Metabolism Disorders and Coagulation Disorders Take Part in Metabolic Disorders Caused by Cd Poisoning in the Uteri of ICR Mice in Labor

KEGG enrichment analysis was performed using the clusterProfiler package and revealed that oxidative phosphorylation was significantly enriched upon Cd exposure (Figure 2A). We found that genes enriched in the oxidative phosphorylation pathway were *mt-Co2*, *mt-Co3*, *mt-Atp6*, *mt-Atp8*, *mt-Nd3*, and *mt-Nd4l*. In a rat liver mitochondria Cd exposure model, it was found that Cd acts on calcium-dependent and thiol-dependent membrane structural domains. This action affects mitochondrial respiration and inhibits oxidative phosphorylation processes [70]. Similarly, Liu et al. reported that in a silkworm midgut cadmium exposure model, the oxidative phosphorylation pathway was significantly enriched in KEGG; the findings are consistent with ours [71]. In addition, our GO enrichment analysis also used the clusterProfiler package, and we found significant enrichment terms, including the aerobic electron transport chain, ATP synthesis-coupled proton transporter, and respirasome (Figure 3B); in these terms, we found that the enriched genes also included *mt-Co2*, *mt-Co3*, *mt-Atp6*, *mt-Atp8*, *mt-Nd3*, and *mt-Nd4l*. The above enrichment analysis results indicate that Cd exposure affected ATP synthesis and led to the disruption of energy metabolism.

In addition, KEGG analysis revealed that complement and coagulation cascades were significantly enriched upon Cd exposure. The enriched genes in complement and coagulation cascades were *Serpind1*, *Fgg*, *Kng1*, *Masp2,* and *Vsig4*. Previous studies have shown that Cd causes coagulation abnormalities, which usually manifest as a hypercoagulable state in the body [72,73]. According to the report by Hara et al., Cd exposure inhibited the fibrinolytic system and led to thrombosis [74]. For pregnant women, Cd exposure can lead to venous thromboembolism [75]. Similarly, in a study about the effect of the metal thallium (Ti) on zebrafish embryonic development, the complement and coagulation cascades were found to be significantly enriched in KEGG, which is similar to our findings [69].

### 4.3. PPI Network Analysis Further Confirmed That Mitochondrial Energy Metabolism Disorders and Coagulation Disorders Were Important Molecular Mechanisms of Cd-Caused Metabolic Disorders in the Uteri of ICR Mice in Labor

We constructed a metabolic mechanism network through a PPI network analysis of all 76 metabolically related DEGs and identified hub genes in the metabolic network by the MCC algorithm, and the visualization results created by the Cytoscape software show that these hub genes formed two clusters (Figure 5B). Notably, the mitochondrial genes *mt-Co2*, *mt-Co3*, *mt-Atp6*, *mt-Atp8*, *mt-Nd3*, and *mt-Nd4l* formed a cluster and exhibited the highest MCC scores (mean = 120.67) (Supplementary File S5), suggesting that mitochondrial energy metabolism is an important molecular mechanism affected by Cd toxicity. The mitochondrial genes *mt-Nd3* and *mt-Nd4l* have been reported to be core structural subunits of respiratory chain Complex I [23,24], while *mt-Co2* and *mt-Co3*, as catalytic subunits of Complex IV (cytochrome c oxidase), are functionally involved in ATP synthesis-coupled electron transfer and mitochondrial electron transport [27,28]. Additionally, *mt-Atp6* and *mt-Atp8* play critical roles in regulating the synthesis and functional assembly of mitochondrial ATP synthase subunits, essential for proton translocation and rotational catalysis [30,31]. In the present study, we observed downregulation of all the above mitochondrial genes (Supplementary File S2), suggesting that Cd induction primarily affects electron transfer for oxidative phosphorylation in mitochondria, leading to abnormal ATP synthesis. Several studies have confirmed our findings. For example, Sokolova et al. investigated the effects of Cd exposure on mitochondrial energy metabolism in *Crassostrea virginica*; they found that Cd exposure caused a 15% reduction in the expression of mRNAs encoding the cytochrome c oxidase (COIV) enzyme in *Crassostrea virginica*, resulting in impaired ATP synthesis [76]. In another example, Al Kaddissi et al. found that crayfish that lived in water containing 10 μg/L of CdCl_2_ for 30 days exhibited a suppression of mRNA expression of *mt-atp6* in the liver and gills [77]. It is noteworthy that our study provides the first evidence that Cd exposure leads to the downregulation of mitochondrial genes (such as *mt-Co2*, *mt-Atp6*, etc.) in the uteri of mice in labor; this evidence provides a new reference for the molecular mechanism of Cd reproductive toxicity and warrants further investigation.

The PPI result visualization contains another cluster of hub genes that were directly involved in the coagulation process. These genes included *Serpind1*, *Fgg*, and *Kng1* (Figure 5B). Although *Serpind1*, *Fgg*, and *Kng1* showed lower MCC scores (13, 25, and 14, respectively) compared to mitochondrial genes (Supplementary File S5), their association with coagulation pathways still supports the notion that coagulation dysfunction is a key mechanism of Cd reproductive toxicity. In this study, we observed that Cd exposure resulted in the upregulation of *Serpind1*, *Fgg*, and *Kng1* in the uterus during labor (Supplementary File S2). *Fgg* is one of the structural genes encoding plasma fibrinogen, which stops bleeding by being converted to fibrin to form blood clots [41]. Nasiadek et al. found a positive correlation between blood Cd concentration and fibrinogen levels in women’s plasma through Spearman correlation analysis [35]; this is consistent with what we observed. *Serpind1* is responsible for encoding antithrombin III during coagulation and can reduce the risk of thrombosis by inhibiting thrombin activity [38]. In a follow-up investigation of 110 patients with acute myocardial infarction, elevated expression of *Serpind1* was found to reduce the risk of atherosclerotic thrombosis [78]. *Kng1* encodes high-molecular-weight kininogen (HK), involved in coagulation regulation, by binding to factor XI (FXI) [79]. It has been reported that a loss of the mouse kininogen gene (Kng1) can lead to the loss of plasma kininogen and delay of thrombosis, and the knockout of *Kng1* can lead to prolonged coagulation time in mouse arteries [44]. To sum up, the expression of the above three genes was upregulated, and through our enrichment analysis and PPI analysis, it can be preliminarily concluded that Cd caused coagulation dysfunction, but the specific mechanisms still need to be further investigated. It is noteworthy that, to date, there are no reports about the relationship between heavy metal exposure and the abnormal expression of *Serpind1* and *Kng1*; however, our current study provides valuable information.

## 5. Conclusions

This study is the first to establish a model of cadmium (Cd) exposure in the uteri of laboring mice, and the study results indicate that Cd exposure leads to energy metabolism disorder by inhibiting the expression of mitochondrial energy metabolism-related genes, while triggering coagulation dysfunction by activating coagulation regulation-related genes in the uteri of mice in labor. Together, impaired energy metabolism and coagulation dysfunction constitute a key mechanism of Cd toxicity in the uteri of ICR mice in labor. These findings not only provide new molecular targets and a theoretical basis for the analysis of Cd reproductive toxicity but also lay an important foundation for human reproductive health research.

## Figures and Tables

**Table 1:** Summary of the sequencing data. Q20: percentage of bases with a Phred value > 20; Q30: percentage of bases with a Phred value > 30. GC (%): percentage of bases G and C in the total number of bases.

Sample	Raw Reads	Clean Reads	Q20 Rate (%)	Q30 Rate (%)	GC Content (%)
CT1	80,294,094	78,488,568	98.75	96.43	48.45
CT2	62,142,356	60,731,292	98.7	96.29	49.08
CT3	74,052,894	72,426,350	98.76	96.44	49.36
Cd1	74,803,466	74,798,244	99.08	96.95	49.89
Cd2	89,375,270	89,369,498	99.15	97.16	48.78
Cd3	87,522,738	87,516,872	98.91	96.48	49.53

**Table 2:** PANTHER enrichment of DEGs in uteri of mice in labor under Cd exposure.

Pathway	ID	*p*-Value	Input
Cytoskeletal regulation by Rho GTPase	P00016	0.037541	*Myh6*, *Myh7*, *Tubb1*
Insulin/IGF pathway–mitogen-activated protein kinase kinase/MAP kinase cascade	P00032	0.041579	*Igf2*, *Irs3*
Nicotinic acetylcholine receptor signaling pathway	P00044	0.046343	*Myo16*, *Myh6*, *Myh7*

## Data Availability

The original contributions presented in this study are included in the article/Supplementary Material. Further inquiries can be directed to the corresponding author(s).

## Supplementary Materials

The following supporting information can be downloaded at: https://www.mdpi.com/article/10.3390/metabo15050339/s1, Supplementary File S1: Transcriptome identified genes data; Supplementary File S2: DEGs in the Cd group versus the CT group; Supplementary File S3: All significantly enriched GO terms; Supplementary File S4: All significantly enriched Reactome pathways; Supplementary File S5: The top 20 genes ranked by MCC scores.
